# Nurse-Led Consultation and Symptom Burden in Patients with Head and Neck Cancer: A Comparative Analysis of Routine Clinical Data

**DOI:** 10.3390/cancers14051227

**Published:** 2022-02-26

**Authors:** Xhyljeta Luta, Sara Colomer-Lahiguera, Rodrigo Jose Martins Cardoso, Frank Hof, Manon Savoie, Cosette Schuler, Justine Wicht, Nadia Fucina, Patricia Debarge, Françoise Ninane, Jean Bourhis, Manuela Eicher

**Affiliations:** 1 Institute of Higher Education and Research in Healthcare, Faculty of Biology and Medicine, Lausanne University Hospital, University of Lausanne, CH-1010 Lausanne, Switzerland; sara.colomer-lahiguera@chuv.ch (S.C.-L.); manon.savoie@chuv.ch (M.S.); justine.wicht@santesarine.ch (J.W.); manuela.eicher@chuv.ch (M.E.); 2 Department of Radiation Oncology, Lausanne University Hospital, CH-1205 Lausanne, Switzerland; rodrigo.cardoso@chuv.ch (R.J.M.C.); francoise.ninane@chuv.ch (F.N.); jean.bourhis@chuv.ch (J.B.); 3 Care Directorate, University Hospitals of Geneva, CH-1211 Geneva, Switzerland; frank.hof@hcuge.ch; 4 Department of Oncology, Lausanne University Hospital, CH-1011 Lausanne, Switzerland; cosette.schuler@chuv.ch (C.S.); patricia.debarge@chuv.ch (P.D.); 5 Care Directorate, Lausanne University Hospital, CH-1011 Lausanne, Switzerland; nadia.fucina@chuv.ch

**Keywords:** head and neck cancer-related symptoms, cancer rehabilitation, radiotherapy, patient-reported outcomes

## Abstract

**Simple Summary:**

During radiotherapy, many patients with head and neck cancer (HNC) experience distressing symptoms that might impact their health-related quality of life (HRQoL) and their ability to cope with the treatment. The objective of this study was to analyze the implementation of nurse-led consultation and the potential associations with symptom burden in HNC patients. Our study included 134 patients, of whom 72 received routine care and 62 received nurse-led consultations. The study was conducted at Lausanne University Hospital between 2017 and 2019. A larger proportion of patients in the routine care group reported severe symptoms; however, no relevant differences in main symptom burden over time were observed between the two groups. Nurse-led consultation has not yet been fully implemented and might be further investigated, involving larger populations, a more detailed process evaluation of the implementation, and the evaluation of the long-term impact of the intervention.

**Abstract:**

Background: Head and neck cancer (HNC) patients experience distressing symptoms that can significantly impact their health-related quality of life (HRQoL). We analyzed the implementation of a nurse-led consultation (NLC) and explored potential associations with symptom burden in HNC patients. Methods: We retrospectively analyzed routinely collected data to describe the implementation of the nurse-led interventions and the evolution of the M.D. Anderson Symptom Inventory scores as patient-reported outcome measures (PROMs). Patients who received routine care (*n* = 72) were compared with patients in the NLC group (*n* = 62) at a radiation oncology unit between 2017 and 2019. PROMs were measured at T0 (between simulation and the first week of radiotherapy), T1 (week 3–4), and T2 (week 5–6). Results: Screening for nutrition, smoking, oral cavity status, and capacity for swallowing/chewing, but not for pain, was applied in >80% of patients in the NLC group from T0 to T1. Education (16%) and care coordination (7%) were implemented to a lesser extent. Symptom burden increased over time with no significant differences between groups. Conclusions: The nurse-led consultation was not associated with symptom burden over time. A larger implementation study including a detailed process evaluation, larger sample size, and a focus on long-term effects is needed.

## 1. Introduction

Head and neck cancer (HNC) is the sixth most prevalent cancer worldwide, with over 800,000 new cases diagnosed and nearly 500,000 deaths each year [1]. It includes malignant tumors of the hypopharynx, oropharynx, lip, oral cavity, nasopharynx, or larynx [2]. Oral cancer is the eleventh most common cancer globally, with well-established major risk factors of tobacco, areca nut, and alcohol consumption, and high-risk human papillomavirus (HR-HPV) types 16 and 18. HR-HPV16/18 are the etiologic agents of cervical cancers and a proportion of oropharyngeal cancers. HPV-associated oropharyngeal and oral cancers show a better prognosis and response to therapy. The potential for the use of de-intensified therapy and prophylactic prevention in HPV-positive oral cancer patients is highlighted [3]. It is widely recognized that alcohol consumption, smoking [4], and, more recently, human papilloma virus (HPV) are the main risk factors for this disease [5]. Radiotherapy, along with surgical excision and/or chemotherapy (depending on tumor extension, comorbidity, and patient preferences), is the standard treatment for HNC patients [6,7]. It has been demonstrated that for T1–T2, the resolutive treatment to date remains surgery, which is, among other things, thanks to minimally invasive technological innovations using the robotic approach [8]. However, the treatment depends on the stage and site of the tumor. Approximately 30–40% of patients with HNC present at an early stage of disease (with stage I or II). These patients are treated with either primary surgery or radiation therapy (RT), whereas patients with carcinoma in situ are usually managed surgically in the same way as those with T0 disease [9].

Despite satisfactory outcomes, many patients experience distressing symptoms that can significantly influence their health-related quality of life (HRQoL), as well as their ability to cope effectively with the treatment [10,11,12,13] and disease-related symptoms. The most frequently reported symptoms are pain, fatigue, poor appetite, diarrhea, dry mouth, shortness of breath, and/or disturbed sleep [12,13,14]. Previous research demonstrates that a high proportion (about 50%) of patients with HNC experience one or more moderate-to-severe systemic symptoms, with prevalence rates ranging from 25% to 40% [15]. Moreover, between 60% and 70% of HNC patients experience moderate-to-severe symptoms, with the highest symptom burden reported among those receiving radiation [16].

Given the complexity of the diagnosis and treatment of the disease, including the necessity of a coordinated follow-up after initial treatment, an integrated care approach for HNC patients is required [17]. In addition to the need for education and self-management support in recurrence prevention, the high prevalence and intensity of symptoms calls for the reinforcement of the symptom management of HNC patients. Among other activities, nurses of HNC patients usually perform screening, assessments, education, support, and care coordination related to mucositis prevention [18,19], radiodermatitis [20], and malnutrition [21,22]. A nurse-led aftercare intervention supporting HNC patients recovering from treatment was highly appreciated by HNC patients. Although the intervention met the need for support in recovery after treatment, it did not improve patients’ HRQoL or self-management skills [23].

The systematic assessment of physical symptoms and psychosocial burden in cancer patients is increasingly used during clinical visits as part of follow-up care [24,25,26,27,28,29]. In that regard, the use of patient-reported outcome measures (PROMs) to monitor symptoms and toxic effects during radiotherapy has been associated with an improvement in patients’ HRQoL and survival and a decrease in hospital admissions [30]. Combining some of these aforementioned assessments and interventions, nurse-led consultations showed positive effects on HRQoL in HNC patients [31]. Nevertheless, the current evidence on the efficacy of nurse-led consultation for HNC remains limited.

In 2017, a nurse-led consultation for HNC patients was developed in a Radiotherapy Outpatient Clinic at the Lausanne University Hospital. A workgroup composed of nurses, physicians, radiotherapy technicians, and nutritionists discussed the contents and scope of the nurse-led consultation, with the goal of optimizing patient care and interdisciplinary collaboration.

The main objective of this study was to describe the implementation of nurse-led consultation and potential associations with symptom burden in HNC patients. Specifically, we analyzed the content of the nurse-led consultation and compared PROMs collected during routine care with the PROMs of patients who received nurse-led consultations in addition to routine care using the M.D. Anderson Symptom Inventory Head and Neck Module (MDASI-HN) [32] three times during radiotherapy treatment.

## 2. Materials and Methods

### 2.1. Study Design and Setting

This is a retrospective analysis of routinely collected data. The study was conducted at the Department of Radiation Oncology, Lausanne University Hospital (CHUV). Before March 2018, the center provided routine nursing care to HNC patients to address specific clinical assessments, education, and information provision. Since March 2018, the center has provided a nurse-led consultation for HNC patients during their radiotherapy treatment with a focus on symptom assessment, the evaluation of risk factors, patient education, and care coordination.

### 2.2. Study Population

Study participants who met the following criteria were included in the study: (1) 18 years or older, (2) diagnosed with HNC, (3) underwent radiotherapy for at least 5 weeks, (4) the first group (routine care) received routine care during their radiotherapy treatment and the second group (nurse-led consultation) received consultation in addition to routine care, and (5) fluent in French.

### 2.3. Data Collection

Data were collected between March 2017 and March 2018 for the routine care group and between March 2018 and June 2019 for the group who received nurse-led consultation in addition to standard care.

### 2.4. Routine Care

Routine care included weekly medical follow-up and standard nursing care. Nurses were available on a daily basis and provided care for patients presenting side effects due to treatment, provided education for self-care, and referred patients to other professionals when needed. Nursing care was provided based on patient request or if other healthcare professionals (e.g., radiotherapy technicians or physicians) identified a complication including sore mouth, difficulty swallowing, fatigue, appetite, skin rash/burning, and weight loss.

### 2.5. Nurse-Led Consultation

The nurse-led consultation aimed to support patients to manage the physical, psychological and social consequences of their disease and its treatment by providing information, emotional support, and education and by coordinating care with other health team members. This consultation was based on the study by Wells et al. [33] and McMillan’s Cancer Support framework, which includes a holistic needs assessment, a personalized care and support plan (in collaboration with the patient), and the organization of health and wellbeing events [34,35]. The main features of these interventions were then combined with the organization already in place at the radiation oncology outpatient clinic. The workgroup discussed HNC patient care and needs to date and developed the nurse-led consultation accordingly.

The first consultation took place between the simulation and the first week of radiotherapy and lasted around 40 min. It included the patient’s history, screening for the risk of malnutrition (with Kondrup score and weight), and smoking status (with daily consumption and attitude towards smoking cessation). The consultation also included the assessment of symptoms such as pain in the ear, nose, and throat sphere; the use of analgesics; the condition of the oral cavity; and the ability to swallow/chew. During these consultations, patients were offered education on preventive measures (mouth washing with baking soda, smoking cessation, diet). When necessary, other professionals from the institution (dietitian, tobacco cessation specialist, speech therapist, radiation oncologist, maxillofacial surgeon) were contacted for further assessment.

The second consultation was conducted between the third and fourth weeks of radiotherapy and lasted about 30 min. It focused on adherence to care (oral care, evolution of smoking), the evaluation of signs and symptoms (weight, pain, mouth condition, and ability to swallow/chew), and the coordination of care by identifying care partners in order to ensure follow-up or contact. In addition, patients in need were screened and assessed for further referral to another member of the interdisciplinary team (HNC specialist, nutritionist or speech therapist).

The third consultation took place at the end of treatment (between the fifth and sixth weeks of radiotherapy) and lasted about 30 min. It focused on the assessment of adherence to care (oral care and smoking), the evaluation of signs and symptoms (weight, pain, oral condition, and ability to swallow and chew), education on warning signs after treatment has ended, and the coordination of care by identifying care partners. The patients were not necessarily followed by the same nurse at each consultation.

All nurses that led the consultation were trained by a clinical nurse specialist in regard to the screening process, information, education materials, and care coordination. It was planned that patients would receive a maximum of 3 consultations of 30–45 min over a period of 6–7 weeks, corresponding to the duration of the radiotherapy treatment. It was agreed that the third consultation should be provided to patients with at least one of the following criteria: age of 75 years and above, cognitive impairment, concomitant treatment (chemotherapy and immunotherapy), or the development of mucositis grade 3. A weekly multidisciplinary meeting (1 h) was also setup with the radiation oncologist, the oncologist, the radiation oncology nurse, the nutritionist/dietitian, and the nurse specialized in nutrition, allowing for the integrated discussion of new and current patients.

### 2.6. Data Collection

The data used to analyze the implementation of the nurse-led consultation were extracted by one of the co-authors (M.S.) from the electronic patient records based on a code-book developed for this study (available upon request). Extraction was validated by a second co-author (J.W.).

We collected PROMs using the M.D. Anderson Symptom Inventory Head and Neck Module (MDASI-HN), which is a reliable and valid instrument designed to measure HNC symptom burden, and symptom interference with daily life [32]. The MDASI-HN covers major symptoms that might cause burden to patients, including core cancer symptoms, HNC-specific symptoms, and MDASI interference items. Patients were asked to rate the severity during the last 24 h of 13 general symptoms, 9 HNC-specific symptoms, and 6 interference items on a scale ranging from 0 (best) to 10 (worst).

Study participants reported PROMs during their course of radiotherapy at three different times: between the simulation and the first week of radiotherapy (T0); between the third and fourth weeks of radiotherapy (T1); and between the fifth and sixth weeks of radiotherapy (T2). We obtained socio-demographic factors (e.g., age, gender, and marital status), behavior (smoking and alcohol), and clinical characteristics (e.g., tumor stage, location, recurrence, and treatment regimen) from electronic medical records.

### 2.7. Statistical Analysis

First, we conducted descriptive statistics to summarize the patient sociodemographic and clinical characteristics. We described continuous variables with mean and standard deviation (SD), and categorical variables with counts and percentages. We categorized symptom severity into three main groups: mild (1–3), moderate (4–6), and severe (7–10). We used linear mixed model regression to compare the longitudinal trajectories of outcomes (symptoms) between the two groups. We corrected for T0 values (first measurement, T0, and two follow-up measurements, T1 and T2). These models included random effects at the level identified by group variable ID (i.e., patient level). Our mixed models were fitted with group, time, and group × time interaction terms. Data management and analysis was conducted using Stata version 16 (StataCorp, College Station, TX, USA).

### 2.8. Ethics

This study was approved by Cantonal Ethics Committee Vaud CER-VD (project number: 2018-01095).

## 3. Results

**Figure 1:**
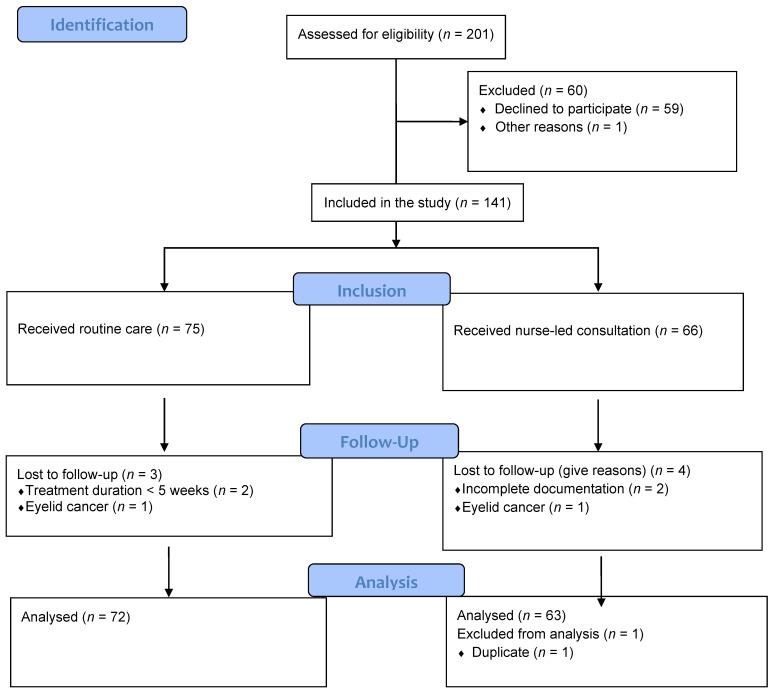
Participant enrolment process.

In total, 201 patients (107 in the routine group and 94 in the nurse consultation group) were screened for eligibility between March 2017 and June 2019 (Figure 1). Of these, 141 were eligible (*n* = 75 in the routine care group and *n* = 66 in the nurse-led consultation group) for participation. Finally, 134 participants (72 in the routine care group and 62 in the nurse-led consultation group) were included in the analysis.

### 3.1. Participant Demographic and Clinical Characteristics

The demographic and clinical characteristics of the study population are presented in Table 1. The overall mean age of study participants was 63.6 (SD, 11.5) years 63.7 (SD, 11.0) in the routine care group and 63.6 (SD, 12.1) in the nurse-led consultation group) and did not differ significantly between the two groups. Our sample was predominately male in both groups (76.4% in the routine care group and 75.8 in the nurse consultation group). The proportion of pre-existing conditions (88.9%) was higher in the routine care group compared with the nurse-led consultation group (62.9%), and this was statistically significant (*p* = 0.0001). The two groups did not differ significantly regarding pre-existing oncological diseases. Patients who received nurse-led consultations were more likely to have cancer recurrence (22.5%) compared with the routine care group (9.7%).

Over half of the patients in the nurse-led consultation group (53.2%) had oropharynx cancer, compared with 37.5% in the routine care group. The proportion of oral cavity (20.8%), larynx (15.3%), and hypopharynx (15.3%) cancer was also higher in the routine care group compared with the nurse consultation group (1.6%, 4.8%, and 8.0%, respectively). About 77% of patients in the nurse-led consultation group underwent tube placement compared with 59% in the routine care group (*p* = 0.02). The two groups did not differ significantly regarding other demographic and clinical variables, including gender, marital status, alcohol consumption, smoking, type of treatment, and hospitalizations.

### 3.2. Nurse-Led Consultations

A description of the nursing consultations is provided in Table 2 and Supplementary Material Figure S1. All the patients were screened to assess the nutritional status at the start of the intervention: 98.4% at the second follow-up and over 97% at the end of radiotherapy treatment compared with education (T0: 58%, T1: 17.5%, and T2: 29.3%) and care coordination (T0: 30.2%, T1: 20.6%, and T2: 22%). Nearly 97% of the patients were screened for smoking status at T0 compared with 93.7% at T1 and 83% at T2. Education for smoking was provided to 15.9% of patients at T0 and 5% at T1, and 6.4% were provided care coordination at T0 and 1.6% at T1.

Approximately 70% of patients were screened for pain at T0 compared with 65.1% at T1 and 63% at T2. Education for pain was provided to 4.8% at T0 and increased to 25% at T1 and 26% at T2. Approximately 6.3% of patients were provided care coordination at T0 and 2.4% at T2. Over 95% of patients received screening for oral cavity status at T0 and 93.7% at T1. Similarly, education was provided to 95.2% of patients at T0 and 73% at T1. Screening for swallowing/chewing capacity was provided to 90.5% of patients at T0 and 81% at T1. Approximately 10% of patients were provided care coordination at T0 and 4.8% at T1.

Three types of intervention (screening, education, and/or coordination) were provided based on the five variables evaluated during the consultations (nutrition, smoking, pain, oral cavity status, and swallowing/chewing capacity). Overall, screening was the most conducted intervention, performed in more than 80% of cases at each of the three different time points, for nutrition and smoking (Table 2 and Figure S1). Education was mostly performed during the first and second consultations, in 16% and 10% of cases, respectively. Coordination with other professionals was performed during first consultation (7%) and 2% during second consultation.

Table 3 provides details on the data collection during nurse-led consultations according to the intervention domain. The median weight at T0 was 70 kg (IQR: 19.9) and decreased to 67.4 (IQR: 14.8) at the end of intervention. The percentage of participants who were screened for nutrition risk (Kondrup) at the start of the intervention was 90.5%. Over 20% had a score of ≥3 (median: 3.0, IQR: 1.0) at T0 and were referred to dieticians for assessment. A total of 46 patients (73%) were reported to be non-smokers during the first consultation, whereas 23.8% were smokers. The proportion of smokers decreased over time. During the first consultation, 33% of smokers were reported to be ambivalent and 47% were motivated to quit smoking during the first consultation, whereas 23.8% of those who smoked reported being active smokers.

**Figure 2:**
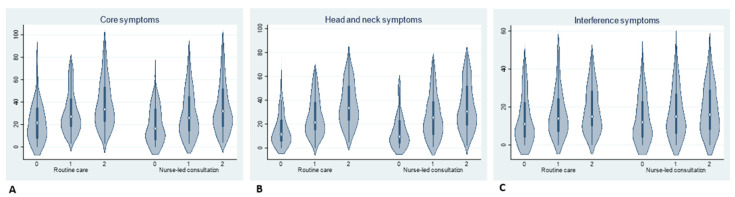
Distribution of data in the routine care and consultation group at three different times across the main MDASI-HN dimensions: core symptoms (**A**); head and neck symptoms (**B**); and interference symptoms (**C**).

The distribution of the data in the routine care and consultation group at three different times and across the main MDASI-HN dimensions is shown in Figure 2.

The raw mean scores for the changes in symptoms for each item on the MDASI-HN at baseline and two follow-up visits by treatment group are shown in Table 4 and Supplementary Material Table S1. Mean scores and baseline-adjusted differences of MDASI-HN by treatment group are shown in Table 4, and raw mean scores are shown in Supplementary Material Table S2.

In the last week of radiotherapy treatment (T2), patients who received nurse-led consultation in addition to routine care had better mean scores for pain compared with the routine care group (4.0 vs. 4.5). The differences between the two groups were small. Though not statistically significant, patients receiving nurse-led consultation in addition to routine care had better mean scores regarding other core symptoms, including fatigue (4.6 vs. 4.9) and appetite (3.7 vs. 4.3) mean, compared with the routine care group.

At the end of radiotherapy treatment, the mean score for difficulty with swallowing/chewing in the nurse-led consultation group was 4.6, compared with 5.3 in the intervention group, as well as tasting food (5.2 vs. 5.8) and burning/rash (3.9 vs. 4.4). Regarding interference symptoms, the nurse-led consultation group had lower mean scores for walking (1.7 vs. 2.7) compared with the routine care group.

**Figure 3:**
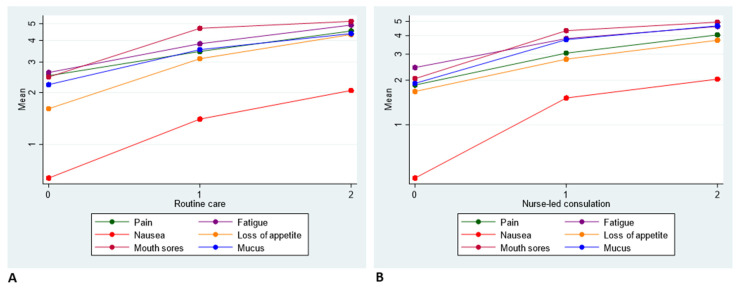
Change in symptoms in the routine care (**A**) and consultation group (**B**) at three different times during radiotherapy treatement.

For both groups, the scores for pain, fatigue, nausea, loss of appetite, mouth sores and mucus worsened over time and in the same direction independently of the group (Figure 3).

A detailed description of the distribution of results according to symptom severity is shown in Supplementary Material Table S2. Some differences in symptom severity were apparent at the end of treatment (T2) between patients in the routine care group and patients followed with nurse-led consultation. These include severe pain and fatigue (30% vs. 24%), drowsiness (20.8% vs. 11%), moderate–severe problems with appetite (25% vs. 19%), and severe burning/rash (33% vs. 20%)

## 4. Discussion

In this study, we evaluated the implementation of a nurse-led consultation and its potential association with symptom burden in HNC patients compared with routine care. A larger proportion of patients in the routine care group reported severe problems related to pain, fatigue, drowsiness, coughing/choking, burning/rash, walking, and appetite. Our mixed models showed no statistically significant differences, except for walking, which showed an improvement in the group receiving nurse-led consultations. We furthermore analyzed the content of the nurse-led consultation. Even if measured at an early stage of implementation, we found a good adherence overall to the screening and assessment of patients over the course of three consultations, with pain assessment being applied in approximately 70% of patients. Educational interventions and the referral of patients were provided and activated to a certain extent, but our results show that these interventions were not always applied for patients with a detected need.

Our study revealed a significant effect of time—the adjusted mean levels for pain, fatigue, nausea, appetite, mouth sores, and mucus increased over time for both groups. This could be due to a steady increase in radiation doses, with the effects gradually rising to eventually attain a plateau, which may vary from patient to patient.

There are several potential reasons why this intervention did not show statistically significant improvements in the nurse-led consultation group. It is possible that the routine oncology care already addressed these relevant needs for both groups. Another important issue might be that PROMs were not directly fed back to nurses and physicians. Thus, nurses planned the interventions based on their evaluations and not necessarily on the results of the MDASI-HN questionnaire. This might have limited their ability to address assessment, educational and referral needs appropriately. Additionally, it is possible that focusing merely on education and coordination and not offering supplemental interventions to reduce symptoms may not show sufficient additional benefit. In addition, as is typical in a university hospital environment, the nursing and physician team composition varied during the implementation year (2018–2019). This may have impacted the implementation of the nurse-led consultation and individual variability in documenting the applied interventions. Patients might have needed more time and more frequent visits to identify and address diverse care needs. Initially, we planned to provide one consultation per week (i.e., 6–7), but due to limited human resources, we were only able to provide a maximum of three consultations per patient. Furthermore, nurses may have struggled to provide consultation as planned due to time constraints, as well as the complex needs of patients. The metastatic disease of the head and neck district occurs in up to 20% of patients, with important repercussions on the patient’s prognosis. The treatment used is certainly more aggressive, which affects the patient’s quality of life [36,37]. There is evidence from previous research showing that nurses encounter challenges in delivering interventions to patients with complex needs, even if they feel fully trained [38]. Another explanation for finding no evidence of the benefits of the nurse-led consultation may be related to the timing of the intervention evaluation. It is possible that our measurements took place too early in the implementation process. Previous research has shown that early assessment may lead to incorrect conclusions about the impact of the interventions [39].

To date, evidence supporting the benefit of nurse-led consultation in HNC patients is sparse. To our knowledge, this is the first evaluation of the implementation of the nurse-led consultation using the validated MDASI-HN multi-symptom inventory. A comparative analysis of a register study [40] analyzed treatment-related changes in patient-reported symptom burden in oropharyngeal squamous cell cancer patients treated surgically and non-surgically using MDASI questionnaire and the EQ-5D health status assessment at four different time points. The authors reported an improvement in symptom burden and QOL after treatment over time regardless of whether patients received surgical or nonsurgical treatment. Another study evaluated the symptom burden for HNC patients using the MDASI-HN questionnaire comparing the group receiving couple-based intervention with that receiving routine medical care. The authors reported less severe symptoms in the intervention group than those in the routine care group [41]. Our findings are in line with previous studies showing an increase in pain scores over time during radiotherapy treatment [16,17,18,19,20,21,22,23,24,25,26,27,28,29,30,31,32,33,34,35,36,37,38,39,40,41,42].

Another strength of this study is the use of mixed models, which allowed the modeling of longitudinal data. Moreover, the development of the nursing consultation allows standards of care (evidence-based guidelines) to be followed, and the paradigm of care to be changed for HNC patients receiving radiotherapy. Nurse-led consultation allowed a standardization and systematization of the care for these patients. While all patients are currently evaluated and screened at baseline and followed during radiotherapy treatment, before the introduction of the nurse-led consultation, only patients with severe symptoms were addressed for specific management. Since the establishment of nurse-led consultation, all patients have had access to information and education (self-care and self-management) and not only in the case of complications. Furthermore, the implementation of the intervention allowed the identification of key players involved in the care of HNC patients receiving radiotherapy, the definition of their roles and responsibilities, the implementation of regular meetings with other professionals, communication and information sharing in a more frequent and systematic manner, and the anticipation (prevention) of problems and complications.

This study has several limitations. First, a relatively small number of patients were included in each group, and this did not allow for a more detailed analysis of the effect of intervention. Therefore, the findings of this study need to be interpreted with caution. Second, we conducted this study at a single center, which may limit the generalizability of our results to other care settings and populations. Third, we used routinely collected health data that were not collected for research purposes. Fourth, we lack detailed information regarding the process of the consultations. Finally, the dependent variables of the study were limited to routinely collected PROMs. To understand the impact of the nurse-led consultation on patient experience and quality of life, it would have been useful to integrate other mediating or moderating variables of symptom burden.

## 5. Conclusions

The implementation of a nurse-led consultation was not associated with a statistically significant improvement in symptom burden in HNC patients. While both groups did not show statistically significant improvements in core symptoms between the start and end of radiotherapy, the nurse-led consultation group had lower (better) mean scores with regard to some symptoms (pain, fatigue appetite) compared with the routine care group at T1 and T2. Our findings reveal that symptoms persist, and some of them even increase over time. This highlights the importance of continued efforts to identify successful interventions that aim at improving symptom burden in HNC patients.

Further research with larger sample sizes is needed to explore the effect of such nurse-led interventions. Based on these results, it is important to evaluate the content of the consultation, and adjust it based on the needs of the patients if necessary. A further study focusing on the long-term effects of the intervention is warranted. Finally, the process evaluation of nurse-led interventions seems to be essential for further research in order to understand the contextual factors that might have impacted the results of this study.

## Figures and Tables

**Table 1:** Demographic and clinical characteristics of patients by treatment group.

Characteristics	Routine Care	NLC	*p*-Value
Age (mean, SD)	63.7 (11.0)	63.5 (12.0)	0.62
Gender *N* (%) Male Female	55 (76.4) 17 (23.6)	47 (75.8) 15 (24.2)	0.93
Marital status *N* (%)			0.39
Married	39 (54.2)	29 (46.7)	
Not married	33 (45.8)	33 (53.2)	
Alcohol *N* (%) consumption			0.50
Yes	33 (45.8)	32 (51.6)	
No	39 (54.2)	30 (48.4)	
Smoking *N* (%)			
Yes	26 (36.1)	14 (22.6)	0.08
No	46 (63.9)	48 (77.4)	
Weight before radiotherapy (kg), mean (SD)	72.6 (13.9)	71.7 (14.9)	0.29
Pre-existing conditions *N* (%)			<0.0001
Yes	64 (88.9)	39 (62.9)	
No	8 (11.1)	23 (37.1)	
Pre-existing oncological conditions *N* (%)			<0.000
Yes	21 (29.2)	20 (32.3)	
No	51 (70.8)	42 (67.7)	
Cancer recurrence *N* (%)			0.04
Yes	7 (9.7)	14 (22.6)	
No	65 (90.3)	48 (77.4)	
Cancer location *N* (%)			<0.000
Oropharynx	27 (37.5)	33 (53.2)	
Oral cavity	15 (20.8)	1 (1.6)	
Nasopharynx	2 (2.8)	4 (6.4)	
Larynx	11 (15.3)	3 (4.8)	
Hypopharynx	11 (15.3)	3 (4.8)	
Salivary glands	4 (5.6)	8 (12.9)	
Nasal cavity	0 (0.0)	7 (11.3)	
Thyroid	2 (2.8)	1 (1.6)	
Stage *N* (%)			0.14
I	3 (4.2)	4 (6.4)	
II	9 (12.5)	9 (14.5)	
III	17 (23.6)	13 (20.9	
IV	42 (58.3)	29 (46.7)	
Unknown	1 (1.4)	7 (11.3)	
Treatment type *N* (%)			0.38
Radiotherapy only	6 (8.3)	8 (12.9)	
Radiotherapy and other	66 (91.6)	54 (87.1)	
Radiotherapy sessions (mean, SD)	32.2 (1.4)	32.5 (1.3)	0.29
Radiation dose (Gy, mean SD)	63.6 (6.8)	64.4 (6.7)	0.68
Radiotherapy interruption			0.68
Yes	12 (16.7)	12 (19.3)	
No	60 (83.3)	50 (80.6)	
Tube placement during radiotherapy			0.02
Yes	29 (59.7)	14 (77.4)	
No	43 (40.3)	48 (22.6)	
Hospitalization during radiotherapy			0.37
Yes	12 (16.7)	7 (11.3)	
No	60 (83.3)	55 (88.7)	
Total	72 (100.0)	62 (100.0)	

NLC: nurse-led consultation.

**Table 2:** Number of nursing interventions provided, according to the intervention domain during the consultations at three different times during radiotherapy treatment.

Consultations		T0 *N* = 63	T1 *N* = 63	T2 *N* = 41 *
Variables	Interventions	*n* (%)	*n* (%)	*n* (%)
Nutrition	Screening	63 (100)	62 (98.4)	40 (97.6)
	Education	37 (58.7)	11 (17.5)	12 (29.3)
	Coordination	19 (30.2)	13 (20.6)	9 (22.0)
Smoking	Screening	61 (96.8)	59 (93.7)	34 (83.0)
	Education	10 (15.9)	6 (9.5)	0
	Coordination	4 (6.4)	1 (1.6)	0
Pain	Screening	44 (69.8)	41 (65.1)	26 (63.4)
	Education	3 (4.8)	16 (25.4)	11 (26.8)
	Coordination	4 (6.3)	0	1 (2.4)
Oral cavity status	Screening	60 (95.2)	59 (93.7)	NA
	Education	60 (95.2)	46 (73.0)	NA
	Coordination	0	0	NA
Swallowing/chewing capacity	Screening	57 (90.5)	51(81.0)	NA
	Education	0	0	NA
	Coordination	6 (9.5)	3 (4.8)	NA

* *N* = 41 patients who met the criteria for the third consultation. T0 = nursing consultation (new case) between the location of scan and the first week of radiotherapy. T1 = follow-up nurse consultation between the third and the fourth weeks of radiotherapy. T2 = nursing consultation (end of treatment) between the fifth and sixth weeks of radiotherapy. NA: not applicable (not reported).

**Table 3:** Results of the data collection during nurse-led consultations.

Variables		T0 *N* = 62		T1 *N* = 62		T2 *N* = 41	
		*n* (%)	MED (IQR)	*n* (%)	MED (IQR)	*n* (%)	MED (IQR)
Nutrition							
Weight	Screening	62 (100)		62 (98.4)		40 (97.6)	
	Kg		70 (19.9)		67.2 (17.4)		67.4 (14.8)
Kondrup ^a^	Screening	57 (90.5)	2.0 (1.0)	NA	NA	NA	NA
	Kondrup ≥ 3	13 (20.6)	3.0 (1.0)				
Smoking	Screening	61 (96.8)		59 (93.7)		34 (83.0)	
Status	Non-smoker	46 (73.0)		48 (76.2)		32 (78.1)	
	Smoker	15 (23.8)		11 (17.5)		2 (4.9)	
Attitude ^b^	Ambivalent	5 (33.0)		3 (27.0)		1 (50.0)	
	Motivated	7 (47.0)		4 (36.0)		1 (50.0)	
Consumption	Screening	11(73.0)		10 (91.0)		2 (100)	
	*n*/day ^c^		20 (21)		10.0 (10)		25 (10)
Pain	Screening	44 (69.8)		41 (65.1)		26 (63.4)	
	Score EVA ^d^		0 (3.0)		2.0 (4.0)		3.0 (5.0)
Oral status	Screening	60 (95.2)		59 (93.7)		NA	NA
	Score OAG ^e^		1.5 (3.0)		3.0 (3.0)		
Swallowing/chewing capacity (dysphagia)	Screening	57 (90.5)		51 (81.0)		NA	NA
	Absence	45 (71.4)		38 (60.3)			
	Presence	5 (7.9)		9 (14.3)			
	Dysphagia associated with other symptoms ^f^	7 (11.1)		4 (6.4)			

T0 = between third and fourth weeks of radiotherapy, T2 = between fifth and sixth weeks of radiotherapy, MED = median, IQR = interquartile range, ^a^: Kondrup nutrition risk score values possible between 0 and 5; ^b^: attitude towards smoking cessation (refers to smokers only); ^c^: number of cigarettes per day (refers to smokers only); ^d^: VAS with possible values between 0 and 10; ^e^: Oral Assessment Guide (OAG) with possible values ranging between 1 and 16; and ^f^: drooling, coughing, change of voice after meals, and accumulation of food in the mouth.

**Table 4:** M.D. Anderson HN mean scores and baseline-adjusted differences for routine care group (RC) and nurse-led intervention group (NLC).

MDASI-HN Dimensions	T1 (3rd–4th Week)				T2 (5th–6th Week)			
	Mean (RC)	Mean (NLC)	Mean Difference (95% CI)	*p*-Value	Mean (RC)	Mean (NLC)	Mean Difference (95% CI)	*p*-Value
Core symptoms								
Pain	3.4	3.0	0.40 (−1.35–0.53)	0.39	4.5	4.0	−0.49 (−1.45–0.46)	0.31
Fatigue	3.8	3.8	−0.01 (−0.84–0.82)	0.98	4.9	4.6	−0.30 (−1.13–0.52)	0.47
Nausea	1.4	1.5	0.11 (0.66–0.88)	0.77	2.0	2.0	−0.02 (−0.94–0.89)	0.96
Disturbed sleep	2.3	2.7	0.37 (−0.55–1.29)	0.43	2.5	2.8	0.24 (−0.63–1.12)	0.58
Being distressed (worried)	2.7	2.9	0.18 (−0.72–1.09)	0.69	2.8	2.9	0.13 (−0.78–1.04)	0.77
Shortness of breath	1.9	1.9	0.06 (−0.75–0.88)	0.87	2.0	1.9	−0.09 (−0.90–0.70)	0.81
Difficulty remembering	2.7	2.9	0.18 (−0.72–1.09)	0.69	2.8	2.9	0.13 (−0.78–1.04)	0.77
Lack of appetite	3.1	2.7	−0.36 (−1.37–0.64)	0.47	4.3	3.7	−0.60 (−1.64–0.43)	0.25
Drowsiness	2.3	2.2	−0.07 (−0.84–0.70)	0.85	3.2	2.9	−0.24 (−1.17–0.68)	0.60
Dry mouth	4.7	4.3	0.23 (−1.27–0.81)	0.45	5.1	4.9	−0.23 (−1.27–0.81)	0.66
Sadness	2.5	2.9	0.41 (−0.58–1.39)	0.41	2.7	2.9	0.18 (−0.84–1.20)	0.72
Vomiting	0.6	0.7	0.02 (−0.55–0.61)	0.92	1.5	1.0	−0.43 (−1.25–0.38)	0.29
Numbness/tingling	1.1	1.5	0.42 (−0.24–1.10)	0.21	1.7	1.8	0.12 (−0.75–1.00)	0.77
Head and neck symptoms								
Mucus	3.5	3.7	0.21 (−0.78–1.21)	0.67	4.4	4.6	0.27 (−0.68–1.22)	0.57
Difficulty with swallowing/chewing	4.4	3.8	−0.57 (−1.61–0.46)	0.28	5.3	4.6	−0.63 (−1.69–0.42)	0.24
Coughing/choking	1.7	1.6	0.32 (−1.30–0.65)	0.86	1.6	1.7	−0.32 (−1.30–0.65)	0.51
Difficulty with voice/speech	3.1	3.2	0.07 (−0.86–1.02)	0.87	3.9	3.9	−0.02 (−1.05–1.01)	0.96
Burning/rash	2.5	2.3	0.52 (−1.56–0.50	0.31	4.4	3.9	−0.52 (−1.56–0.50)	0.31
Constipation	1.8	2.2	0.37 (−0.58–1.33)	0.63	2.7	2.4	−0.24 (−1.25–0.77)	0.63
Problem with tasting food	4.0	3.7	−0.37 (−1.55–0.79)	0.53	5.8	5.1	−0.64 (−1.83–0.54)	0.28
Mouth/throat sores	3.4	3.5	0.11 (−0.97–1.20)	0.83	4.4	4.3	−0.03 (−1.13–1.06)	0.94
Problem with teeth or gums	2.3	3.0	0.69 (−0.27–1.67)	0.16	2.8	3.0	0.20 (−0.80–1.21)	0.69
Interference symptoms								
General activity	3.4	3.4	0.07 (−0.88–1.03)	0.87	4.0	4.1	0.09 (−0.88–1.07)	0.84
Mood	2.9	3.3	0.36 (−0.61–1.33)	0.46	3.5	3.3	0.36 (−0.61–1.33)	0.46
Work	3.8	3.4	−0.46 (−1.58–0.64)	0.41	3.7	4.0	0.35 (−0.72–1.42)	0.52
Relationships with others	2.4	2.3	−0.12 (−0.98–0.73)	0.77	2.8	2.7	−0.11 (−0.98–0.75)	0.79
Walking	2.2	2.1	−0.17 (−1.09–0.75)	0.71	2.7	1.7	−0.96 (−1.85–0.08)	0.03
Joy of living	2.6	3.5	0.93 (−0.08–1.95)	0.07	2.6	3.3	0.66 (−0.35–1.67)	0.20

## Data Availability

The data presented in this study are available on request from the corresponding author. The data are not publicly available due to privacy and ethical restrictions.

## Supplementary Materials

The following are available online: https://www.mdpi.com/article/10.3390/cancers14051227/s1. Figure S1: Number and type of nursing interventions during radiotherapy treatment; Table S1: Raw MD-HN mean scores by treatment group across three different times. Table S2: Distribution of symptoms according to severity.
