# Supplementary material for: Nurse-Led Consultation and Symptom Burden in Patients with Head and Neck Cancer: A Comparative Analysis of Routine Clinical Data
Source: Cancers (Basel). 2022 Feb 26;14(5):1227. doi: 10.3390/cancers14051227 (PMC8909718; doi:10.3390/cancers14051227)
Supplement: Supplementary file 1 [file cancers-14-01227-s001.zip › cancers-1602142-supplementary.pdf]

cancers

Article

Nurse-Led Consultation and Symptom Burden in Patients with Head and Neck Cancer: A Comparative Analysis of Routine Clinical Data

Supplementary Materials:

Figure S1: Number and type of nursing interventions during radiotherapy treatment;
Table S1: Raw MD-HN mean scores by treatment group across three different times.
Table S2: Distribution of symptoms according to severity.

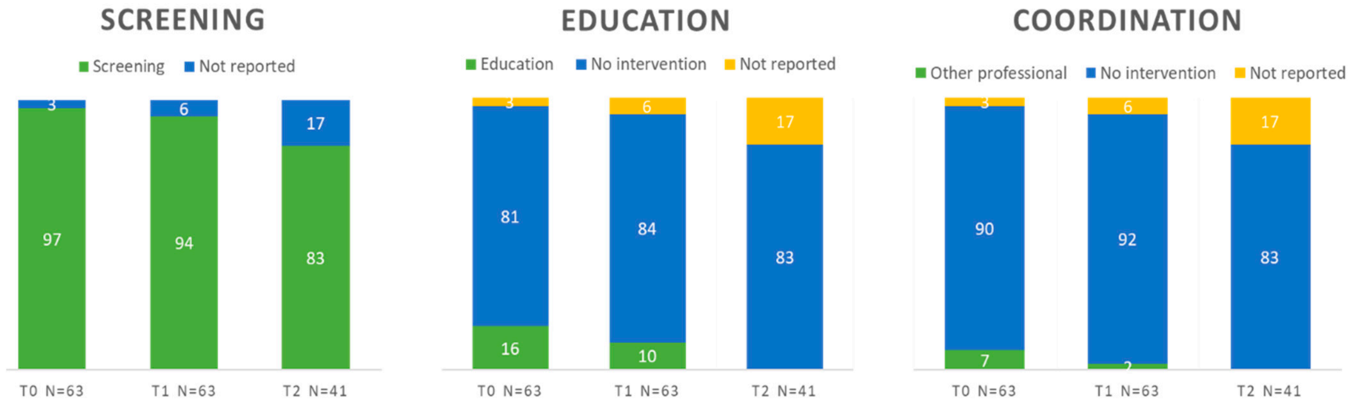

Figure S1. Number and type of nursing interventions during radiotherapy treatment.

Table S1. Raw MD-HN mean scores by treatment group across three time points.

	**Routine Care**						**Routine Care + Nurse Consultation**					
**Period**	**T0**		**T2**		**T3**		**T0**		**T2**		**T3**	
**Core Symptoms (Mean. Median. IQR)**	**Mean score**	**Median (IQR)**	**Mean**	**Median (IQR)**	**Mean**	**Median (IQR)**	**Mean**	**Median (IQR)**	**Mean**	**Median (IQR)**	**Mean**	**Median (IQR)**
Pain	2.5	1 (5)	3.4	3 (4.5)	4.5	4.5 (4)	1.8	1 (3)	3	3(3)	4	3 (4)
Fatigue	2.6	2 (5)	3.8	3.5 (4)	4.9	5 (4)	2.4	2 (4)	3.8	3(3)	4.6	4.5 (3)
Nausea	0.6	0 (0)	1.4	0 (3)	2	1(3.5)	0.4	0 (0)	1.5	0 (2)	2	1 (3)
Disturbed sleep	2.4	1.5 (3)	2.3	1(4)	2.5	2(5)	2.3	2 (5)	2.6	2 (4)	2.7	2(2)
Being distressed (worried)	2.8	2 (5)	2.7	2 (4)	2.8	2 (4.5)	2.9	2 (5)	2.9	2 (4)	2.9	2 (4)
Shortness of breath	1.6	0.5 (2)	1.9	1 (3)	2	1 (3)	1.3	0(2)	1.9	1 (3)	1.9	1 (3)
Difficulty remembering	1.4	0 (1.5)	1.4	0 (2)	1.7	1(3)	1.2	0 (1)	1.4	1 (2)	1.4	0.5 (2)
Lack of appetite	1.6	0 (3)	3.1	2.5 (5)	4.3	4 (4.5)	1.7	0 (3)	2.7	2 (4)	3.7	3 (5)
Drowsiness	1.7	1 (3)	2.3	2 (4)	3.2	2 (5.5)	1.4	0 (2)	2.2	2 (3)	2.9	2 (4)
Dry mouth	2.4	1 (4)	4.7	5 (5)	5.2	5 (5)	2	1 (3)	4.3	4 (5)	4.9	5 (6)
Sadness	2.5	2 (4)	2.5	2 (4)	2.7	2 (4)	2.3	1 (5)	2.9	2 (4)	2.9	2 (3)
Vomiting	0.6	0 (0)	0.6	0 (0.5)	1.5	0 (2)	0.2	0 (0)	0.7	0 (0)	1	0 (1)
Numbness/tingling	1.6	0 (2)	1.1	0.5 (2)	1.7	1 (3)	1.1	0 (2)	1.5	1 (2)	1.8	0 (2)
**Head and neck symptoms**												
Mucus	2.2	1 (4)	3.5	3 (5)	4.4	4 (4.5)	1.9	1 (4)	3.7	3 (5)	4.6	4 (4)
Difficulty with swallowing/chewing	3	2 (5)	4.4	4 (5)	5.3	5 (5)	2.5	1 (5)	3.8	3.5 (4)	4.6	4 (5)
Coughing/choking	1.8	1 (3)	1.7	1 (3)	3	2 (5)	1.2	0 (2)	1.6	1 (2)	2.7	2 (5)
Difficulty with voice/speech	2.6	2 (5)	3	2 (4)	3.9	4 (4.5)	2.5	2 (4)	3.2	2 (5)	3.9	3 (5)
Burning/rash	1.2	0 (2)	2.5	2 (4.5)	4.4	4 (5.5)	0.9	0 (1)	2.3	1.5 (4)	3.9	3.5 (5)
Constipation	1	0 (1)	1.8	0 (3)	2.7	2 (4.5)	1.4	0 (2)	2.2	1 (3)	2.4	1.5 (3)
Problem with tasting food	1.4	0 (2)	4	3 (6)	5.8	6 (6)	1.6	0(2)	3.7	2.5 (5)	5.2	5 (7)
Mouth/throat sores	1.7	1 (2.5)	3.4	2 (6)	4.4	4 (5.5)	1.7	0 (3)	3.5	2 (5)	4.3	4 (6)
Problem with teeth or gums	1.5	0 (2)	2.3	1 (4)	2.8	2 (5)	1.9	0 (3)	3	2 (5)	3	2 (4)
**Interference symptoms**												
General activity	2.6	2 (5)	3.4	3 (4)	4	4 (4)	3	2.5 (5)	3.4	3 (4)	4.1	3 (4)
Work	2.9	2 (6)	3.8	3 (7)	3.6	3 (5)	3.1	2 (5)	3.4	3 (5)	4	4 (6)
Walking	2	1 (4)	2.2	1 (4)	2.7	2 (5)	1.7	0 (3)	2	1 (3)	1.7	1 (2)

Table S2. Distribution of symptoms according to severity.

	Routine Care			Nurse Consultations		
**Period**	**T0**	**T1**	**T2**	**T0**	**T1**	**T2**
**Core symptoms**	%	%	%	%	%	%
**Pain**						
Mild	73.6	66.7	50	85.5	75.8	67.7
Moderate	8.3	13.9	18.1	4.8	12.9	8.1
Severe	18.1	19.4	31.9	9.7	11.3	24.2
**Fatigue**						
Mild	73.6	62.5	40.3	80.6	62.9	50
Moderate	18.1	19.4	29.2	9.7	19.3	25.8
Severe	8.3	18.1	30.6	9.7	17.7	24.2
**Nausea**						
Mild	93.1	88.9	77.8	98.4	87.1	83.9
Moderate	5.6	8.3	11.1	1.6	6.4	3.2
Severe	1.3	2.8	11.1	0	6.4	12.9
**Disturbed sleep**						
Mild	77.8	76.4	70.8	70.9	77.4	79
Moderate	8.3	13.9	20.8	24.2	11.3	11.3
Severe	13.9	9.7	8.3	4.8	11.3	9.7
**Being distressed (worried)**						
Mild	70.8	76.4	75	69.3	74.2	72.6
Moderate	19.4	9.7	11.1	16.1	14.5	12.9
Severe	9.7	13.9	13.9	14.5	11.3	14.5
**Shortness of breath**						
Mild	90.3	83.3	84.7	87.1	85.5	82.3
Moderate	—	9.7	8.3	9.7	4.8	8.1
Severe	9.7	6.9	8.3	3.2	9.7	9.7
**Difficulty remembering**						
Mild	88.9	88.9	87.5	90.3	88.7	90.3
Moderate	2.8	2.8	6.9	4.8	6.4	4.8
Severe	8.3	8.3	5.6	4.8	4.8	4.8
**Lack of appetite**						
Mild	86.1	69.4	55.6	85.5	77.4	59.7
Moderate	8.3	15.3	19.4	4.8	8.1	20.9
Severe	5.6	15.3	25	9.8	14.5	19.3
**Drowsiness**						
Mild	81.9	79.2	66.7	88.7	82.3	74.2
Moderate	12.5	12.5	12.5	9.7	11.3	14.5
Severe	5.6	8.3	20.8	1.6	6.4	11.3
**Dry mouth**						
Mild	76.4	48.6	40.3	83.8	58.1	45.2
Moderate	12.5	22.2	20.8	8.1	12.9	16.1
Severe	11.1	29.2	38.9	8.1	29	38.7
**Sadness**						
Mild	76.4	77.8	77.8	74.2	75.8	75.8
Moderate	15.3	9.7	8.3	16.1	6.4	4.8
Severe	8.3	12.5	13.9	9.7	17.7	19.3

**Vomiting**						
Mild	97.2	95.8	86.1	100	91.9	90.3
Moderate	—	4.2	5.6	—	4.8	1.6
Severe	2.8	—	8.3	—	3.2	8.1
**Numbness/tingling**						
Mild	86.1	94.4	91.7	87.1	87.1	80.6
Moderate	6.9	4.2	2.8	11.3	8.1	8
Severe	6.9	1.4	5.6	1.6	4.8	11.3
**Head and neck symptoms**						
**Mucus**						
Mild	80.6	59.7	56.9	79	58.1	51.6
Moderate	6.9	25	18.1	17.7	20.9	19.3
Severe	12.5	15.3	25	3.2	20.9	29
**Difficulty with swallowing/chewing**						
Mild	72.2	58.3	41.7	72.6	66.1	56.4
Moderate	9.7	13.9	18.1	14.5	14.5	12.9
Severe	18.1	27.8	40.3	12.9	19.3	30.6
**Coughing/choking**						
Mild	86.1	90.3	68.1	93.5	88.7	72.6
Moderate	4.2	5.6	12.5	3.2	4.8	12.9
Severe	9.7	4.2	19.4	3.2	6.4	14.5
**Difficulty with voice/speech**						
Mild	73.6	70.8	59.7	77.4	61.3	59.7
Moderate	12.5	16.7	16.7	11.3	20.9	16.1
Severe	13.9	12.5	13.6	11.3	17.7	24.2
**Burning/rash**						
Mild	90.3	75	51.4	95.2	79	62.9
Moderate	5.5	19.4	15.3	1.6	9.7	16.1
Severe	4.2	5.6	33.3	3.2	11.3	20.9
**Constipation**						
Mild	91.7	84.7	75	87.1	83.8	82.3
Moderate	4.2	5.6	8.3	8.1	3.2	3.2
Severe	4.2	9.7	16.7	4.8	12.9	14.5
**Problem with tasting food**						
Mild	88.9	58.3	36.1	83.8	67.7	46.7
Moderate	5.6	13.9	18.1	9.7	8.1	9.7
Severe	5.6	27.8	45.8	6.4	24.2	43.5
**Mouth/throat sores**						
Mild	86.1	65.3	54.2	87.1	64.5	54.8
Moderate	6.9	12.5	15.3	3.2	12.9	16.1
Severe	6.9	22.2	30.6	9.7	22.6	29
**Problem with teeth or gums**	86.1	76.4	72.2	80.6	72.6	69.3
Mild	6.9	8.3	12.5	8.1	9.7	14.5
Moderate	6.9	15.3	15.3	11.3	17.7	16.1
Severe						
**Interference symptoms**						
**General activity**						

Mild	69.4	72.2	52.8	62.9	64.5	59.7
Moderate	18.1	8.3	26.4	16.1	17.7	17.7
Severe	12.5	19.4	20.8	20.9	17.7	22.6
**Work**						
Mild	69.4	65.3	59.7	67.7	59.7	56.4
Moderate	9.7	9.7	23.6	16.1	22.6	17.7
Severe	20.8	25	16.7	16.1	17.7	25.8
**Relations with others**						
Mild	80.6	81.9	75	80.6	75.8	75.8
Moderate	9.7	8.3	15.3	12.9	12.9	16.1
Severe	9.7	9.7	9.7	6.4	6.4	8.1
**Walking**						
Mild	79.2	77.8	73.6	80.6	80.6	85.5
Moderate	13.9	12.5	13.9	9.7	9.7	9.7
Severe	6.9	9.7	12.5	9.7	9.7	4.8
**Joy of living**						
Mild	75	81.9	81.9	77.4	66.1	74.2
Moderate	11.1	4.2	4.2	4.8	12.9	8.1
Severe	13.9	13.9	13.9	17.7	20.9	17.7
